# Predictors of intestinal parasite infection among HIV patients on antiretroviral therapy in Jos, Plateau State, Nigeria, 2016: a cross-sectional survey

**DOI:** 10.11604/pamj.2021.38.306.25751

**Published:** 2021-03-24

**Authors:** Olawunmi Toyin Ajayi, Olufunmilola Bamidele Makanjuola, Adebola Tolulope Olayinka, Abdulhakeem Olorukooba, Josephine Ene Olofu, Patrick Nguku, Olufunmilayo Ibitola Fawole

**Affiliations:** 1 Nigeria Field Epidemiology and Laboratory Training Program, No. 50 Haile Selassie Street, Asokoro, Abuja, Nigeria,; 2 Department of Medical Microbiology and Parasitology, University of Ibadan, Ibadan, Oyo State, Nigeria,; 3 Department of Community Medicine, Ahmadu Bello University, Zaria, Kaduna State, Nigeria,; 4 Federal College of Veterinary and Medical Laboratory Technology, Vom, Jos, Plateau State, Nigeria,; 5 Department of Epidemiology and Medical Statistics, University of Ibadan, Ibadan, Oyo State, Nigeria

**Keywords:** Antiretroviral therapy, HIV/AIDS, prevalence, intestinal parasites, protozoa, factors, toilets

## Abstract

**Introduction:**

intestinal parasitic infection has been reported as a cause of morbidity and mortality among HIV patients on antiretroviral therapy (ART) due to interruption in treatment of the defaulting HIV patients. This study aimed to determine the prevalence and possible causes of intestinal parasites among HIV patients on ART.

**Methods:**

a survey involving 375 adult HIV/AIDS patients selected using a systematic random sampling technique was conducted in a Jos University Teaching hospital, Plateau State, Nigeria. Socio-demographic and clinical data was collected using semi-structured interviewer administered questionnaire and electronic dataset review. Fresh stool samples were collected from all participants for laboratory identification of intestinal parasites using formol-ether sedimentation and modified Ziehl-Neelsen techniques. Descriptive statistics, odds ratio and logistic regression model were computed at P ≤ 0.05.

**Results:**

the mean age of the study participants was 41.6±9.3years. Majority 294 (78.4%) were females, 141 (37.6%) lived in the rural area, 50 (13.3%) respondents did not have toilets in their homes. Most 275 (73.3%) had ART adherence level of 95% and above. Prevalence of intestinal parasites was 28.5%. Females (aOR = 2.14, 95% CI=1.12 – 3.89) and participants with no toilet facilities (aOR = 2.0, 95% CI=1.03 – 3.94) were significantly more likely to have intestinal parasites.

**Conclusion:**

the prevalence of intestinal parasites was high among HIV patients. Gender and unavailability of toilet in homes were found to be predictors of having parasites. We recommend that HIV patients should be periodically screened for IPs during the follow-up clinic visits.

## Introduction

The Human Immunodeficiency Virus (HIV) pandemic is one among the major health challenges ever faced by humanity. Globally, by 2019, 38.0 million people were estimated to have HIV and 690,000 people were reported to have died from HIV related causes. In 2019, 1.7 million people became newly infected with HIV compared with over 2 million in 2015. Also, in 2019 over 25.4 million (67%) people living with HIV (PLHIV) worldwide were on antiretroviral therapy (ART) [1]. In Nigeria, in 2018, about 1.9 million people were living with HIV and it is estimated that 130,000 new HIV infections occurred in that year, while 53,000 persons died from AIDS-related causes [2]. In the absence of ART, HIV/AIDS patients in many low income countries continue to suffer the health consequences of intestinal protozoan as a result of their accompanying low CD4 cell count [3]. In order to improve patient care response, ART was made available to persons living with the virus by the Federal government and some non-governmental organizations [4].

Several intestinal parasites have been incriminated as vital contributors to morbidity among HIV infected persons living in low income countries, and the common parasites include: *Cryptosporidium spp., Isospora belli, Microsporidia spp., Entamoeba histolytica, Giardia intestinalis, Trichuris trichiura, Ascaris lumbricoides, Strongyloides stercoralis*,and hookworm which are also associated with diarrhea and iron deficiency anemia [5]. The occurrence of intestinal parasitic infection is likewise significantly high in sub-Saharan Africa ranging from 50% - 70% among different groups, where the substantial number of HIV/AIDS cases were concentrated. The incidence and prevalence of infection with an enteric parasite in individuals living with HIV/AIDS often depends on the endemicity of that parasite in the community. In general, *Cryptosporidium spp, I. belli* and *E. histolytica* have been reported as the most frequently identified parasites, and these represent a significant cause of diarrhea among HIV/AIDS patients [3, 6]. Most intestinal parasite infection in PLHIV were associated with an increased risk of death, patients with morbidity due to intestinal parasites (IPs) may have interruptions in ART resulting in a rapid disease progression [7]. In addition, low viral load, CD4 count greater than 500, the absence of diarrhea and the ART treatment are not always indicative of free parasitic infection [8].

Parasitic infections in HIV infected patients are common in many regions and populations across Nigeria and this represent a serious public health challenge. Interactions between HIV and other infective agents, including parasites, influence the health status of people living with HIV/AIDS [7]. Intestinal protozoa infections continued to cause morbidity and mortality in HIV/AIDS patients even after they received ART. Mortality often occurs because patients do not have a sustained response to antiretroviral agents. The lack of sustainability occurs because of poor adherence, drug toxicities, drug interactions, or acquisition of a drug-resistant strain abintio [7, 9]. Different intestinal parasitic infections occur in HIV patients and organisms vary from one country to the other. Routine diagnosis of opportunistic intestinal parasites among HIV patients on ART is not usually done at follow up clinic. There is lack of empirical data on the prevalence of intestinal parasites among HIV/AIDS patients on ART. Although studies have determined the prevalence of intestinal parasites among PLHIV in Nigeria [10, 11], information on intestinal parasites and its magnitude among HIV patients on ART is scarce. Furthermore, predictors of occurrence of intestinal parasites among HIV patients on ART were not determined. Therefore, this study aims to determine the prevalence of intestinal parasites and its predictors among HIV patients on ART.

## Methods

**Study area:** the study was conducted at antiretroviral clinic - AIDS Prevention Initiative Nigeria/Jos University Teaching Hospital (APIN/JUTH), Plateau State. APIN/JUTH centre was established in 2001 to provide comprehensive treatment, care and support to PLHIV/AIDS. It provides antiretroviral treatment for adults, children, pregnant women (PMTCT) and laboratory services to monitor CD4 cell count. The clinic also serves as a referral centre for HIV/AIDS patients in north- central region of the country. At the clinic, patients are followed up every three months and an additional follow-up visit is done after two weeks if a patient has commenced on ART. ART is initiated if patients had CD4 counts < 350 cells/mm^3^ or are symptomatic.

**Study design:** the study was hospital based cross-sectional survey.

**Study population:** the study population comprised of adult (> 18 years) HIV/AIDS patients attending APIN/JUTH clinic, Plateau State. Patients who were on antihelminthic or antifungal treatment at the time of recruitment, who had treatment for any parasitic or fungal infections two weeks preceding specimen collection, leaving outside the Plateau State and those who were unable to produce stool samples during clinic hours were excluded from the survey.

**Sample size:** the sample size was calculated using the formula for cross sectional survey, assuming a standard normal deviate of 95% confidence interval and precision level of 5%. The proportion of intestinal parasites among HIV patients on ART was 30.5% [12]. A minimum sample size of 364 was obtained after a 10% adjustment for non-response. Data was collected from 375 respondents.

**Sampling technique:** a systematic random sampling technique was used to select participants. Using the total number of HIV/AIDS patients that attend the clinic per week, based on the estimated sample size, the number of samples to be collected each week was determined. Adjustments were made for patients who may not consent and who may not be able to produce stool. The average number of HIV/AIDS patients that access the clinic for healthcare is 210 patients per week. Based on the estimated sample size and after adjusting for non-response, 75 patients were recruited weekly. Every third adult HIV/AIDS patients that came to the clinic on the day of sample collection between November 2015 to January 2016 were recruited into the study until the required sample size was achieved.

**Study instrument:** data were collected using a semi-structured questionnaire. The study instrument was developed after extensive literature review. The questionnaire was pre-tested at another clinic different from the survey site. The questionnaire had three sections namely: socio-demographic (age, sex, residential area, highest educational status), socio-economic (types of toilet, sources of drinking water) and clinical characteristics (WHO clinical stage, last CD4 count, ART adherence level, presence of diarrhea). The patients´ most recent last CD4 count and WHO clinical stage were obtained from the clinics electronic data base.

**Data collection:** data were collected at APIN/JUTH clinic, Jos, Plateau State by the principal investigator and two recruited research assistants. Data collection were done between November 2015 and January 2016.

**Specimen collection:** approximately two grams of fresh feaces (uncontaminated by urine) was collected into a clean, dry, leak-proof universal bottle. The specimen was transported in a cold chain system and analyzed within 5 - 8 hours of collection. For watery faecal specimens, about 3 mls of feaces was collected which was transported to the laboratory as soon as possible. Formol - ether sedimentation and modified Ziehl - Neelsen techniques was carried out on the freshly collected stool sample for identification of intestinal parasites and opportunistic intestinal protozoa respectively [13].

### Study variables

**Dependent variable:** presence of intestinal parasite in the stool (yes or no).

**Independent variables:** the independent variables were socio-demographic characteristics including: age (20-29, 30-39, 40-49, > 50 years), sex (male, female), education (no education, primary, secondary, tertiary), and residential area (rural, urban), clinical characteristics: WHO clinical stage (I-II, III-IV), CD4 count (≤ 200, 201-350, ≥ 351), ART adherence (< 90, 90-95, ≤ 95), sources of water (borehole, well, pipe borne and stream) and type of toilet (water closet, pit latrine and lack of toilet).

**Data analysis:** data were entered and cleaned using Microsoft Excel 2013, after which they were exported to EPI INFO 7.1.5.2 version software for analysis. Univariate analysis was used to determine frequencies and proportions. Respondent´s socio-demographic, clinical and socio-economic characteristics including the prevalence of intestinal parasites were expressed as frequencies and percentages. Bivariate analysis was also done by computing odds ratio at 95% confidence interval to determine the relationship between presence of intestinal parasites and respondent´s socio-demographic or clinical and/or socio-economic characteristics. The level of statistical significance was set at α < 0.05. Multivariate analysis was done using multiple logistic regression to adjust for the effect of confounders. Predictor factors with P-value < 0.25 in the odds ratio at bivariate analysis were included in the logistic regression model.

**Ethical considerations:** ethical approval was obtained from Jos University Teaching Hospital (JUTH) ethical committee (JUTH/DCS/ADM/127/XIX/6316). Written informed consent was obtained from all the study participants before questionnaire administration and sample collection. The confidentiality and privacy of participants was protected and participation was voluntary. Feedback on results were communicated to the respondents and they were referred to the health facility if in need of treatment.

## Results

The mean age of the respondent was 41.6years SD±9.3. Majorities (78.4%) were females, (58.1%) were married, and (40.5%) had tertiary education while (82.2%) were employed. Most of the participants (63.8%) had been enrolled at the clinic for 6 to 10years while (4.63%) were enrolled in less than a year. The duration uses of ART ranged from 4 months to 15 years, with a median of 8 (5-10) years. The source of drinking water for majority (70.4%) of the participants was pipe borne water. Ninety- three (24.8%) had co-morbidities such as hypertension, hepatitis, peptic ulcer and diabetes. Twenty-one (5.6%) respondents had diarrhea during stool sample collection and 275 (73.3%) had ART adherence level of 95% and above. At the enrolment, 217 (57.9%) patients were in WHO stage I and II. Of the 345 patients with documented last CD4 count, (64.8%) had CD4 count above 351 cells/mm^3^ (Table 1).

**Table 1:** demographic and clinical characteristics of the participants

Demographic/clinical characteristics	Frequency (n = 375)	Percentage (%)
**Age (years)**		
20-29	36	9.6
30-39	120	32.0
40-49	147	39.2
≥50	72	19.2
**Sex**		
Male	81	21.6
Female	294	78.4
**Residential area**		
Rural	141	37.6
Urban	234	62.4
**Highest educational level**		
No education	41	10.9
Primary	65	17.3
Secondary	117	31.2
Tertiary	152	40.6
**No of years since enrolment**		
<1	22	6.7
1-5	77	20.5
6-10	234	62.4
≥ 11	39	10.4
Baseline WHO stage*		
I-II	217	72.1
III-IV	84	27.9
**Types of toilet**		
Water closet	238	63.5
Pit latrine	87	23.2
No toilet	50	13.3
**Main Sources of drinking water**		
Pipe borne	264	70.4
Well	107	28.5
Stream	4	1.1
Last CD4 count(cells/mm3)*		
≤ 200	36	9.6
201-350	66	17.6
≥ 351	243	64.8
**ART adherence level (%)**		
< 90	50	13.3
90-94	50	13.3
≥ 95	275	73.4
**Co-morbidities**		
Yes	93	24.8
No	282	75.2
**Presence of diarrhea**		
Yes	21	5.6
No	354	94.4

* Not add up to sample size due to missing value

**Figure 1:**
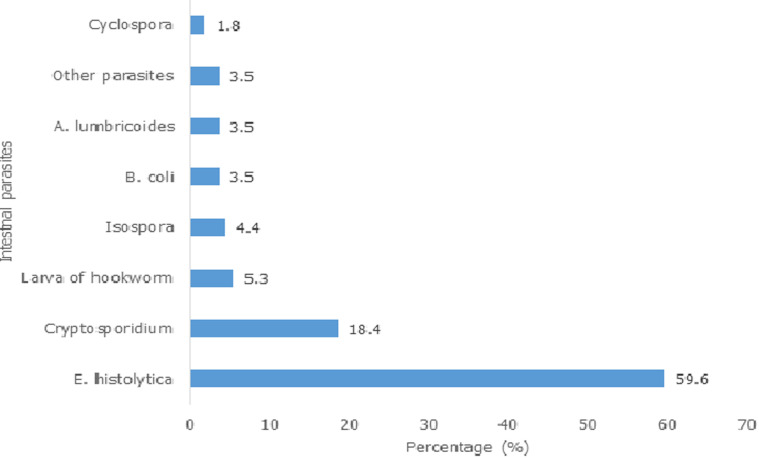
prevalence of intestinal parasites among HIV patients

A total of 375 stool samples were examined for intestinal parasites. One hundred and seven participants had at least one positive intestinal parasites giving an overall prevalence of 28.5%. The overall prevalence of intestinal protozoa identified among the intestinal parasites was 24.6% of which 18.4% had *Cryptosporidium spp, Isospora* 4.4%, *Cyclospora spp* had the least prevalence of 1.8%. The most prevalent protozoa and helminthes parasites identified among the patients were cyst of *E. histolytica* and larva of hookworm with prevalence of 59.6% and 5.3% respectively (Figure 1).

The socio-demographic variables significantly related with having intestinal parasites included: female sex (OR=2.21, 95% CI=1.18 - 4.14), and living in the rural area (OR=1.80, 95% CI=1.14 - 2.84) were more likely to have intestinal parasites. Although the risk of intestinal parasites was higher among the unemployed (OR= 1.28, 95% CI=0.73 - 2.26), younger aged respondents (age group 30-39 years) (OR=1.24, 95% CI= 0.65 - 2.39) compared to respondents above 50 years of age, the differences were not statistically significant. Patients with no toilet facilities were significantly more likely to have intestinal parasites (OR= 2.44, 95% CI= 1.32 - 4.49) compared with those with toilet facilities in their homes. There was no significant relationship between patients´ source of drinking water and presence of intestinal parasites. In addition, there was no significant association between the participants´ clinical characteristics and occurrence of intestinal parasites (Table 2).

**Table 2:** association between intestinal parasites and socio-demographic/clinical factors

	Intestinal parasites			
Socio-demographic/clinical factors	Present n (%)	Absent n (%)	cOR (95% CI)	P-Value
**Age (years)**				
20-29	10 (27.8)	26 (72.2)	1.07 (0.44 – 2.63)	0.44
30-39	37 (30.8)	83 (69.2)	1.24 (0.65 – 2.39)	0.26
40-49	41 (27.9)	106 (72.1)	1.07 (0.57 – 2.03)	0.94
≥ 50	19 (26.4)	53 (73.6)	1.00	
**Sex**				
Female	93 (31.6)	201 (68.4)	2.21 (1.18 - 4.14)	0.01
Male	14 (17.3)	67 (82.7)	1.00	
**Residence**				
Rural	51 (36.2)	90 (63.8)	1.80 (1.14 - 2.84)	0.01
Urban	56 (23.9)	178 (76.1)	1.00	
**Marital Status**				
Widowed/Divorced	27 (30.7)	61 (69.3)	1.08 (0.63 – 1.87)	0.86
Single	17 (24.6)	52 (75.4)	0.80 (0.42 – 1.49)	0.59
Married	63 (28.9)	155 (71.1)	1.00	
**Occupation**				
Unemployed	22 (32.8)	45 (67.2)	1.28 (0.73 – 2.26)	0.48
Employed	85(27.6)	223 (72.4)	1.00	
**Source of drinking water**				
Borehole and Pipe borne	73 (27.7)	191 (72.3)	0.87 (0.53 – 1.41)	0.65
Well and Stream	34 (30.6)	77 (69.4)	1.00	
**Types of toilet**				
No toilet	23 (46.0)	43 (86.0)	2.44 (1.32 – 4.49)	0.003
Water closet/Pit	84 (25.8)	241 (74.2)	1.00	
**Baseline WHO stage**				
I-II	64 (28.7)	159 (71.3)	0.98 (0.56 - 1.69)	0.95
III-IV	25 (29.1)	61 (70.9)	1.00	
**Last CD4 count (cells/mm3)**				
≥ 351	65 (26.7)	178 (73.3)	1.09 (0.49 - 2.45)	0.82
201 - 350	25 (37.9)	41 (62.1)	1.83 (0.74 - 4.52)	0.19
≤ 200	9 (25.0)	27 (75.0)	1.00	
**ART adherence level (%)**				
≥ 95	79 (28.7)	196 (71.3)	1.14 (0.57 - 2.27)	0.69
90 - 94	15 (30.0)	35 (70.0)	1.22 (0.50 - 2.93)	0.66
< 90	13 (26.0)	37 (74.0)	1.00	
**Presence of Diarrhea**				
Yes	0 (0.0)	21 (100.0)	Undefined	
No	107 (30.2)	247 (69.8)	1.00	
**Comorbidities**				
Yes	30 (32.3)	63 (67.7)	1.27 (0.76 – 2.11)	0.36
No	77 (27.3)	205 (72.7)	1.00	

cOR = crude odds ratio

The variables that were significant at 95% CI at bivariate analysis namely sex, residential area and lack of toilet facilities were included in the multivariate analysis. The risk factors identified for the development of opportunistic intestinal parasites after adjusting for confounders were female sex (aOR = 2.14 (1.12 - 3.89) and lack of toilet facilities (aOR= 2.01 (1.03 - 3.94). Females and respondents with no toilets in their homes are twice more likely to have intestinal parasites than their male counterpart and those with toilet facilities (Table 3).

**Table 3:** risk factors associated with intestinal parasites among HIV patients

Risk factors	cOR (95% CI)		aOR(95%CI)	P-value
**Sex**				
Female	2.21 (1.18 – 4.14)	0.01	2.14 (1.12 – 3.89)	0.02
Male	1.00		1.00	
**Residence**				
Rural	1.80 (1.14 – 2.84)	0.01	1.40 (0.77-2.34)	0.17
Urban	1.00		1.00	
**Types of toilet**				
No toilet	2.44 (1.32 – 4.49)	0.003	2.01 (1.03 – 3.94)	0.04
Water closet/pit	1.00		1.00	

cOR = crude odds ratio; aOR = adjusted odds ratio; CI = confidence intervals

## Discussion

This study found intestinal parasites prevalence among HIV patients on ART of 28.5%. This finding is consistent with previous studies conducted in Ethiopia [14], Cameroon [15, 16] and India [17], where similar prevalence were recorded among HIV patients on ART. However, our findings reveal a lower prevalence compared with studies reported in Nigeria [18] and Cameroon [19]. The difference reported in Cameroon [19] could be due to different laboratory detection techniques used to ensure no parasites were missed even when present in low quantity. However, our study employed fewer detection methods. Furthermore, a study in Ethiopia reported a higher prevalence which might be due to the study been conducted among ART naive HIV patients [20]. Nevertheless, the prevalence in our study was found to be higher than those reported in Nigeria [11] and India [21], despite the fact that the previous studies were conducted among antiretroviral naive patients compared with our study which was carried out among HIV patients on ART.

Our finding further reported *Cryptosporidium species, I. belli* and *Cyclospora*as the major opportunistic intestinal parasites identified. This is conforming to the work reported in Ethiopia, where *Cryptosporidium, Microsporidium, Cyclospora and Isospora belli* were the commonest opportunistic intestinal parasites reported among HIV-infected persons [20]. Although, it is known that opportunistic intestinal protozoa constituted a major health problem in patients infected with HIV leading to manifestation of diarrhea, however there was no report of diarrhea among the HIV patients who had opportunistic intestinal parasites in our study, this might be due to the high adherence level to ART as reported by the respondents in our study. Nevertheless, intestinal parasites are associated with diarrhea and iron deficiency anemia. Low viral load, CD4 count greater than 500, the absence of diarrhea and the ART treatment are not always indicative of free parasitic infection as found in this and other studies [8]. HIV patients on ART could be predisposed to intestinal parasitic infection due to interruptions in ART [7].

We found *Cryptosporidium* species to be the most prevalent among the opportunistic intestinal parasites. Similar findings have been reported by authors [22-24] in Ethiopia, Lesotho and China, *Cryptosporidium* was found to be the leading opportunistic intestinal parasites among HIV patients and the commonest cause of morbidity and mortality in HIV positive individuals worldwide [25]. However, our finding is inconsistent with studies conducted in India [21, 26] which reported *Isospora spp*. as the leading opportunistic intestinal parasites in PLWHA. This difference may be as a result of different in geographical location. Additionally, the Ethiopian study [22] was among HIV patients with diarrhea disease, while most of the respondents in our study had no diarrhea. *Isospora belli* infection was reportedly common among patients with AIDS and chronic diarrhea in low income countries and was found in 12-19 % of patients with diarrhea disease in Zambia, Haiti and Uganda [27].

The most prevalent protozoan and helminthes identified in our study were cysts of *E. histolytica* and the larva of Hookworm. This was in agreement with studies conducted in Ethiopia [28], Kenya [29] and Nigeria [30], where *E. histolytica* was identified as the most prevalent protozoan parasites among PLWHA who were ART naïve. These findings were inconsistent with the results of another study conducted by [18] in Nigeria, where a higher prevalence of helminthes infections compared with protozoan infections was reported among HIV/AIDS patients on ART. This discordance in the prevalence of *E. histolytica* may be due to variations in the method of parasite detection used.

Intestinal parasitic infections was more common in women, which is in agreement with a studies conducted in Nigeria and Ethiopia [11, 30]. The female preponderance is probably because of women´s engagement in domestic chores and agricultural activities such as child care, washing and contact with contaminated soil more than men [31]. In other studies, male gender was found to be strongly associated with the occurrence of opportunistic infections [32, 33]. The inconsistency in the result as regards sex may be due to the type of opportunistic pathogens studied, our study assessed intestinal parasites among PLHIV while the other studies [33] assessed *M. tuberculosis*.The lack of toilet facilities in the home was significantly associated with intestinal parasites among HIV patients. This finding was supported by a study done in Port Harcourt, Nigeria which showed 50% parasite positive patients did not have a toilet in their homes and by another study in Malaysia which showed indiscriminate defecation was significantly associated with parasitic infections [34, 35]. Additionally, a study conducted in Ethiopia showed a high prevalence of parasite positive HIV/AIDS patients did not have toilet in their homes and they were more likely to have parasitic infections than those with toilets at home [28].

It was observed in our study that, lower CD4 count ≤ 200cells/mm^3^ of the participants was not significantly associated with intestinal parasites infection. This finding was inconsistent with studies conducted in India [36], Cameroon [19] and Ethiopia [16] which showed occurrence of intestinal parasites among HIV patients to be significantly associated with CD4 cell count ≤ 200 cells/mm^3^. This inconsistency may be as a result of the most recent CD4 cell count of the participant used which did not represent the current CD4 count of the participant at the time of sample collection. However, a study reported decreased rate of intestinal parasites among HIV patients on ART with CD4 count ≤ 200 cells/mm^3^ [37] which was in agreement with our study. This may indicate the success of ART intervention in reducing opportunistic infection including intestinal parasites among the HIV high risk group of lower CD4 count.

Our study was subject to some limitations. Some participants could not produce stool samples during follow up at the clinic. Such participants were excluded from the study if they were not residing in Plateau State. Participant´s current CD4 count was obtained by reviewing their clinical records, however due to incomplete data, respondents most recent CD4 count was used instead of their current CD4 count. Furthermore, only one stool specimen was collected from each participant and intestinal parasitic detection was based only on the use of formol ether sedimentation and Zeihl Neelsen staining technique both of which might have led to an under-estimation of intestinal parasites and opportunistic intestinal protozoa respectively.

## Conclusion

The prevalence of intestinal parasites among HIV patients on ART was 28.5%. Female sex and lack of toilet facilities were found to be significantly associated with occurrence of intestinal parasites. Health education on personal hygiene, importance of toilet facilities in the home and adequate treatment when tested positive for intestinal parasites are necessary to reduce or eliminate these parasites. Furthermore, testing for intestinal parasites among HIV patients on ART should be done periodically at follow-up clinics to optimize early detection and treatment.

### What is known about this topic

- Intestinal parasitic infection is common among ART naïve HIV patients with diarrhea disease;
- Intestinal parasitic infection among HIV patients is related to poor adherence to ART level and CD4 count below 200 cells/mm^3^.

### What this study adds

- Intestinal parasitic infection occurs among HIV patients on ART without causing diarrhea disease;
- HIV patients with adherence level greater than 95% and CD4 count above 350 cells/mm^3^ could be infected with intestinal parasites;
- Females with HIV infection are more likely to have intestinal parasites than their male counterpart.
